# Postdecision Evidence Integration and Depressive Symptoms

**DOI:** 10.3389/fpsyt.2019.00639

**Published:** 2019-09-23

**Authors:** Madeleine E. Moses-Payne, Max Rollwage, Stephen M. Fleming, Jonathan P. Roiser

**Affiliations:** ^1^Institute of Cognitive Neuroscience, University College London, London, United Kingdom; ^2^Wellcome Trust Centre for Neuroimaging, University College London, London, United Kingdom; ^3^Department for Imaging Neurosciences, Max Planck University College London Centre for Computational Psychiatry and Ageing Research, London, United Kingdom

**Keywords:** metacognition, depression, self-esteem, decision making, confidence, postdecision evidence

## Abstract

**Background:** Metacognition, or the ability to reflect on one’s own thoughts, may be important in the development of depressive symptoms. Recent work has reported that depressive symptoms were associated with lower metacognitive bias (overall confidence) during perceptual decision making and a trend toward a positive association with metacognitive sensitivity (the ability to discriminate correct and incorrect decisions). Here, we extended this work, investigating whether confidence judgments are more malleable in individuals experiencing depressive symptoms. We hypothesized that depressive symptoms would be associated with greater adjustment of confidence in light of new evidence presented after a perceptual decision had been made.

**Methods:** Participants (N = 416) were recruited *via* Amazon Mechanical Turk. Metacognitive confidence was assessed through two perceptual decision-making tasks. In both tasks, participants made a decision about which of two squares contained more dots. In the first task, participants rated their confidence immediately following the decision, whereas in the second task, participants observed new evidence (always in the same direction as initial evidence) before rating their confidence. Participants also completed questionnaires measuring depressive symptoms and self-esteem.

**Analysis:** Metacognitive bias was calculated as overall mean confidence, whereas metacognitive sensitivity was calculated using meta-d’ (a response-bias free measure of how closely confidence tracks task performance) in the first task. Postdecision evidence integration (PDEI) was defined as the change in confidence following postdecision evidence on the second task.

**Results:** Participants with more depressive symptoms made greater confidence adjustments (i.e., greater PDEI) in light of new evidence (β = 0.119, p = 0.045), confirming our main hypothesis. We also observed that lower overall confidence was associated with greater depressive symptoms, although this narrowly missed statistical significance (β = -0.099, p = 0.056), and we did not find an association between metacognitive sensitivity (meta-d’) and depressive symptoms. Notably, self-esteem was robustly associated with overall confidence (β = 0.203, p < 0.001), which remained significant when controlling for depressive symptoms.

**Conclusions:** We found that individuals with depressive symptoms were more influenced by postdecisional evidence, adjusting their confidence more in light of new evidence. Individuals with low self-esteem were less confident about their initial decisions. This study should be replicated in a clinically depressed sample.

## Background

Individuals have the ability to reflect on and report their mental states. In this way, decisions are usually accompanied by a degree of confidence (or uncertainty) regarding accuracy, which is often termed a metacognitive judgment (1). The ability to accurately track performance with confidence ratings is known as metacognitive ability (2), and this varies substantially among individuals (3).

### Early Investigations Into Metacognition and Depression

The ability to reflect on our thoughts may be important in the development, maintenance of, and recovery from a depressive episode. Early investigations into metacognition in depression focused on self-reinforcement (4, 5). In these studies, participants were asked to evaluate their performance by retrospectively administering self-reward (or self-punishment) by choosing the number of tokens (that translated into monetary reward) they believed they deserved for their performance on various tasks. Depressed patients were consistently less willing to reward, and more willing to punish, themselves (4, 5). However, because self-evaluation in these studies entailed explicit reinforcement, this pattern of results is difficult to interpret. An alternative explanation is that depressed patients in fact believed that they performed as well as nondepressed individuals, but that they did not deserve reward (or deserved punishment) despite good performance. This would align with the well-known tendency for depressed patients to experience excessive feelings of guilt, leading to the belief that they deserve punishment (6, 7).

### Confidence Judgments in Depression

In the late 1970s, the idea of depressive realism was proposed (8), which stimulated further investigation into metacognition in depression. Alloy and Abramson (8) suggested that depressed patients are sadder but wiser, that is, that they hold a more realistic view of themselves and the world compared with healthy individuals who are influenced by a rose-tinted positive bias. This challenged both clinical convention and earlier cognitive models of depression [e.g., Refs. (9, 10)], which focused on the idea that thoughts in depressed patients were dominated by negative schemata perpetuated through negative biases in the processing of new information. By contrast, according to the depressive realism account, healthy participants should show a positive bias, rating their performance more favorably (overconfidence), whereas depressed individuals should report a more accurate account of their performance.

The evidence for the depressive realism hypothesis is mixed, especially when assessed *via* confidence in decision-making paradigms. In these experiments, a metric of calibration is inferred by comparing reported percentage correct (confidence) to actual percentage correct (accuracy). When confidence is rated after the decision task, depressed patients have commonly been found to exhibit pessimistic calibration, being approximately twice as likely to rate their performance below chance compared to healthy controls (11–14). However, such posttest differences in metacognitive bias could be influenced by a negative memory bias. In other designs, confidence ratings are made on a trial-by-trial basis. Depressed participants seem to show a lowered overconfidence effect on such tasks, meaning that their judgments more accurately reflect their long-run performance (11, 15, 16), which would be consistent with depressive realism. However, in some studies, this difference was specific to correct trials only (14, 17) or depended on whether the participant expected to do badly before the test (11), indicating that depressive realism may be context-dependent.

Quiles et al. (18) measured metacognitive awareness (as termed by the authors) using a more sophisticated method—by calculating Hamann’s coefficient (19). This involves creating a contingency table of concordance and disconcordance between performance and confidence scores. Hamann’s coefficient was then used as a measure of metacognitive awareness on four different cognitive tasks. This study detected a positive relationship between metacognitive awareness and depression scores on a facial emotion recognition task (i.e., confidence ratings were more closely aligned with actual performance in depressed individuals). However, there was no evidence of such an association with metacognitive awareness on tests of executive function, digit span, or episodic memory. Quiles et al. (18) also measured self-esteem using Rosenberg’s Self-Esteem Questionnaire (20) but found no association with metacognitive awareness.

One difficulty in interpreting the above findings is that these measures conflate metacognitive bias (the extent to which subjects have the tendency to rate high or low confidence) with metacognitive sensitivity (the ability to discriminate correct from incorrect decisions). However, in theory, the overall level of confidence (metacognitive bias) is independent of the ability to discriminate between correct and incorrect decisions (metacognitive sensitivity) as one can have overall relatively low confidence but still appropriately differentiate between correct and incorrect decisions (i.e., selectively assigning higher confidence to correct decisions). Such concerns have led to novel computational methods to assess metacognitive sensitivity, which helps formalize different facets of metacognition and create more precise evaluations of the bias and sensitivity of metacognitive judgments (2). This dissociation between confidence bias and metacognitive sensitivity has important theoretical implications regarding the association with depression because these two concepts may track different psychological phenomena, that is, general negative self-evaluation versus depressive realism.

### Association Between Metacognitive Sensitivity and Depression

In an effort to tease apart these constructs, Rouault et al. (21) recently utilized computational methods and trial-by-trial confidence ratings to separately measure confidence bias and metacognitive sensitivity, alongside questionnaires assessing various symptoms of mental illness in two large online samples (N = 498 and N = 497). Using factor analysis to cluster symptoms, they found that high scores on questions loading onto a depression/anxiety factor were associated with significantly lower overall confidence in decision making. They also reported a trend-level association with metacognitive efficiency (the level of metacognitive sensitivity expected for a given level of task performance), such that participants with higher depression/anxiety factor scores were better able to discriminate between correct and incorrect trials.

Importantly, Rouault et al. (21) matched performance across all participants using a staircase procedure, meaning that differences in metacognitive judgments could not be explained by poorer performance in participants with high depression/anxiety factor scores. Equating task difficulty is crucial because unless groups are matched for accuracy, it is hard to dissociate metacognitive judgments from performance (because worse performance would be expected to elicit both lower overall confidence and impair trial-by-trial sensitivity) (21).

### Postdecision Evidence Integration and Depressive Symptoms

Metacognitive evaluations have been tightly linked to postdecision evidence processing (22, 23) or the utilization of information not yet available for the decision itself (24). This process of ongoing evidence integration that occurs postdecision is especially important for recognising errors or changing one’s mind (25–27). Thus, recent studies have sought to identify mechanisms supporting postdecision processing (28, 29) and link such mechanisms to metacognitive ability (30, 31). Investigating confidence adjustments based on postdecision evidence represents a natural extension of studies of metacognitive ability.

This might be especially important for our understanding of symptoms of depression, such as indecisiveness (more frequent changes of mind). Indecisiveness is a core symptom of depression, which may be important when considered alongside other interest-activity symptoms as a predictor of antidepressant treatment outcome (32). Previous research has found that dogmatism, which could be considered a rigid decisiveness, is associated with fewer changes of mind (31). Therefore, in the present study, we directly assessed the influence of postdecision evidence on confidence judgments and their relationship to symptoms of depression.

### Role of Self-Esteem in Depression and Metacognition

Low self-esteem or self-worth are common symptoms of depression and play a central role in the classic cognitive models of depression (33). Low self-esteem can also prospectively predict depressive symptoms across the life span (34, 35).

Previous research has investigated the relationship between metacognition and self-efficacy, which is often considered a facet of self-esteem and describes a person’s core beliefs about their ability to produce desired effects in their environment (36). Metacognition and self-efficacy may interact to guide learning. For example, a student’s metacognitive judgment that they have better knowledge of one topic than another in an upcoming exam may lead them to study the latter more intensively. On the other hand, the same student’s self-efficacy judgment about their ability to pass the exam may encourage (if optimistic) or hinder (if pessimistic) their motivation to study the less well-known topic. Such interactions between metacognition and self-esteem have been studied in relation to academic performance (37–39). However, to our knowledge, no previous study has examined the relationship between self-esteem and metacognitive function using cognitive tasks, which was one of the aims of the current study.

### Current Study

In this study, we aimed to extend Rouault et al.’s (21) findings in a new sample of participants using a similar perceptual decision-making paradigm but also including a task that manipulated postdecision evidence. Alongside these two tasks, we measured depressive symptoms and self-esteem. As in Rouault et al. (21), participants were matched for perceptual discrimination performance using a staircase procedure. Therefore, we were able to discriminate between metacognitive bias (the overall degree of confidence), metacognitive sensitivity (the alignment between confidence ratings and accuracy), and postdecision evidence integration [(PDEI) the adjustment of confidence according to information provided after the decision was made].

First, we hypothesized that depressive symptoms would be associated with a lower metacognitive bias (i.e., lower overall confidence in decisions), as reported by Rouault et al. (21). Second, we hypothesized that depressive symptoms would be associated with higher metacognitive sensitivity (better calibration of confidence to accuracy, e.g., higher meta-d’), which did not survive correction for multiple comparisons in Rouault et al. (21). Finally, we hypothesized that depressive symptoms would be associated with a greater sensitivity to postdecision evidence, reflected in a greater increase in confidence following confirmatory evidence (indicating that the participant was correct) and a greater decrease in confidence following disconfirmatory evidence (indicating that the participant wasincorrect). This final variable was our primary outcome of interest and is novel to this experiment. We additionally included a measure of self-esteem to assess whether individual differences in metacognition were associated with this construct, which is highly relevant to depression. (34).

## Methods

### Online Recruitment and Participants

Participants were recruited *via* Amazon Mechanical Turk. Subjects gave informed consent, and the study was approved by the Research Ethics Committee of University College London (study number 1260-003). Participants were paid a basic payment of $4.50 and earned a bonus of up to $3.50 (M = $3.01, SD = $0.22) based on task performance (explained below).

A total of 575 participants took part in the study, and 416 participants’ data were analyzed. Data were collected as part of another project investigating the relationship between PDEI and radical political beliefs, which is reported elsewhere (31). The sample size was based on power calculations conducted in relation to the original politics effects reported in Rollwage et al. (31) because this was the main aim of the study. With N = 416, we had 80% power to detect an effect size (r) of 0.14 between depressive symptoms and metacognitive variables at p = 0.05 (two-tailed).

Out of 416 subjects, 219 were female and 196 were male (one participant selected “rather not say”). The mean age of the participants was 35.85 (range: 18–71 years). Participants reported a range of education levels, from high school to doctoral degree, with most participants having completed college-level education.

Participants had to be 18 years or older and were restricted to the United States and prevented from participating multiple times. Participants were excluded on the following grounds: if they failed to answer at least one of two catch questions presented within the questionnaires (n = 17); their perceptual discrimination performance exceeded 85% or dropped below 60% correct (indicating that the staircase procedure did not converge undermining the validity of the task, n = 90); they chose the same confidence rating more than 90% of the time (indicating that participants may not have engaged with the confidence reports, n = 11); their median confidence rating response time was below 850 ms (indicating a very quick and possibly careless rating, n = 19); they missed over 5% of trials (n = 21); or they missed questions in the depression questionnaire (n = 1). These data were collected as a follow-up to a previous experiment [as explained in Ref. (29)]; thus, these task-based exclusion criteria were defined *a priori* [based on Study 1 in Ref. (31)].

### Overview of Procedure

**Figure 1:**
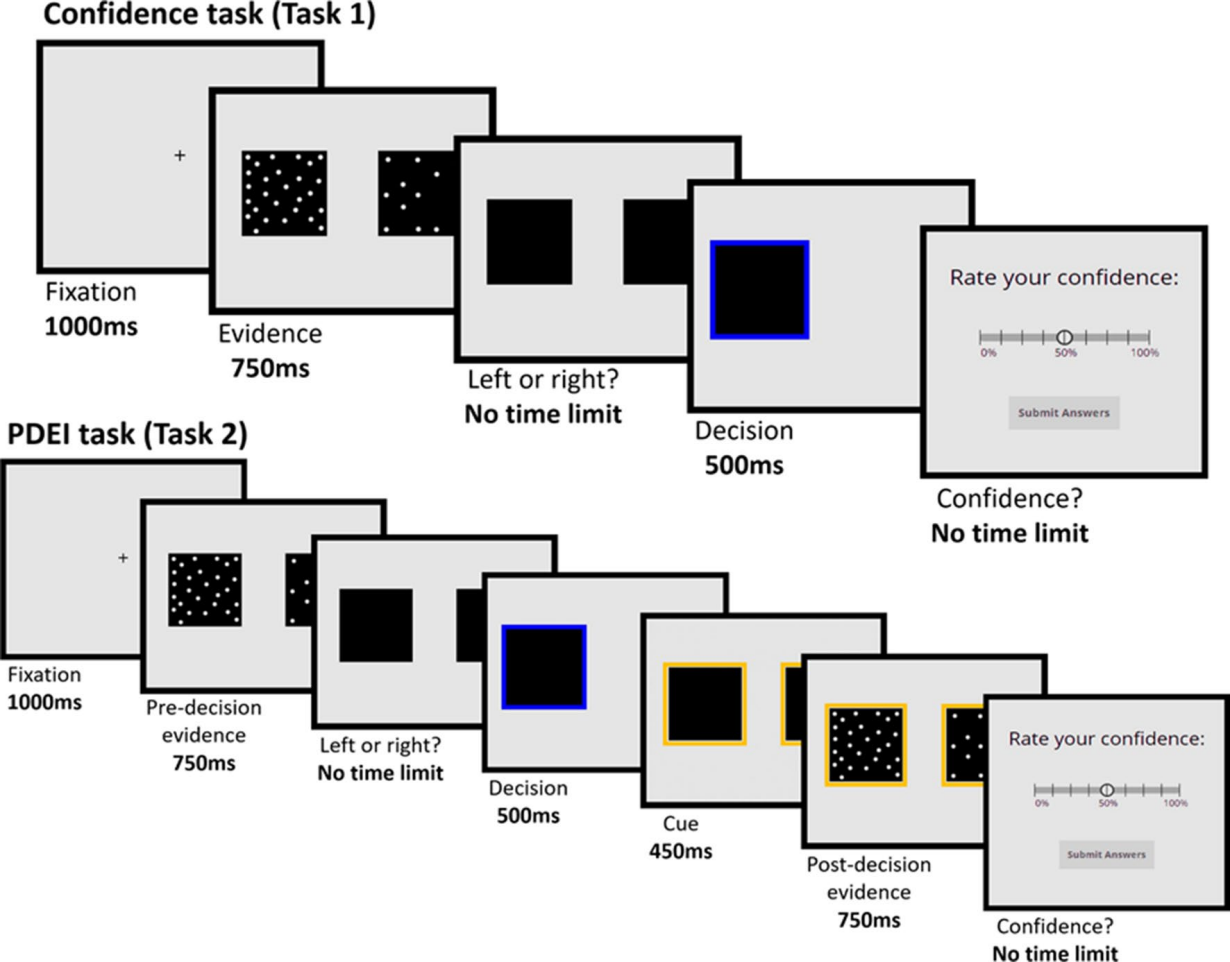
Confidence task (Task 1) and Postdecision Evidence Integration (PDEI) task (Task 2). Participants were asked to judge which of two squares contained more flickering dots. In the Confidence task, participants immediately rated their confidence following their decision. In the PDEI task, participants viewed a new set of dots (always in the same direction as the predecision evidence. Half of trials showed evidence of the same strength as in predecision phase, whereas half of the trials showed evidence of greater strength) before rating their confidence in their original decision.

Participants were given general information and instructions and completed an online consent form. Then, participants completed the calibration phase, which lasted about 10 min. Next, participants completed the confidence task (∼10 min) followed by the PDEI task (∼20–30 min) Please see **Figure 1** for task procedure. Finally, participants completed multiple questionnaires, including the Zung (40) depression questionnaire and the self-esteem rating [results relating to the other questionnaires are reported in Ref. (31)]. In total, participants spent about 60 min completing the experiment.

### Experimental Design

#### Stimuli

Both tasks were programmed in JavaScript and were presented *via* the online platform Gorilla (https://gorilla.sc/). Stimuli for the perceptual decision consisted of two black squares (each 250*250 pixels) presented halfway up the screen, one to the right and one to the left of center. The squares were subdivided into 625 cells, which were randomly selected to be filled with dots. On each trial, one square always contained 313 cells filled with dots, and the other square contained a greater number of cells filled with dots—the exact difference in dot numbers was calibrated to individual participants and determined using a staircase procedure (described below). The configuration of dots (which cells contained/did not contain dots) was created randomly and changed four times during a single trial, with each configuration being presented for 150 ms. This gave the impression of flickering dots. The smaller the difference in dots between the two squares, the more difficult the perceptual decision. Within each trial, the square which contained more dots (left/right) remained constant.

#### Calibration

Performance was matched across participants using a staircase procedure, in which participants judged which of two squares contained more dots, but confidence ratings were not required. This procedure was used to identify the evidence strength (i.e., difference in dots) required to elicit approximately 71% accuracy for each participant. To do this, we used a 2-down–1-up staircase procedure that operates on the logarithm of the difference in the number of dots. Unlike either of the main tasks, during the calibration, participants were not asked for confidence ratings but were given visual feedback for each trial—showing a green frame around the chosen option if they were correct or a red frame around the chosen option if they were incorrect.

The calibration stage consisted of 120 trials. Participants completed 70 trials during the staircase procedure, and the average evidence strength of the last 25 trials was used for the initial decisions throughout the rest of the experiment. A further 50 trials were completed to be used to establish the dot difference for the high postdecision evidence strength trials. In these trials, the logarithm of the difference in the number of dots was multiplied by a factor of 1.3. These trials were interleaved within the other trials but only appeared after 20 “burn-in” trials (to allow the staircase to converge) and were yoked to the concurrent staircase value. This higher evidence strength evoked mean performance levels of 81.45% correct (SD = 10.43%).

#### Confidence Task (Task 1)

The confidence task consisted of 60 trials in total. In each trial, participants were again asked to make a judgment as to which square contained the larger number of dots. After making their decision, participants rated their confidence in their judgment by indicating the probability that their decision was correct. This was done by mouse click on a 9-point sliding scale, with the lowest category labeled 0%, the highest category labeled 100%, and the midpoint labeled 50%.

Participants were incentivized to give accurate confidence ratings through a points system using a quadratic scoring rule (41):

$$\text{point}\text{​}s=100*[1-{\left({\mathrm{correct}}_{i}-{\mathrm{conf}}_{i}\right)}^{2}]$$

where correct_i_ is 1 when the participant is correct on trial i and 0 when they are incorrect, and conf_i_ is the participant’s confidence rating on trial i. This means that to gain maximum points, participants should accurately report their confidence—the most points are earned when one is both maximally confident and correct or minimally confident and incorrect. For every 5,000 points earned, subjects received an extra $1.

#### Postdecision Evidence Integration Task (Task 2)

The PDEI task consisted of 120 trials, 60 with low postdecision evidence strength and 60 with high postdecision evidence strength. As in the confidence task, participants made a judgment as to which square contained more dots. After making this decision, participants were shown an additional sample of flickering dots. In half of the trials, the new sample was of the same strength to the initial sample (low postdecision evidence strength), and in the other half, the evidence was stronger (calibrated at 80% accuracy—high postdecision evidence strength). Participants rated their confidence in their initial decision only after seeing both predecision and postdecision samples. Importantly, the postdecision evidence was always in the same (correct) direction as the predecision evidence. Participants were instructed that the extra evidence was bonus information that could be used to inform their confidence ratings.

### Depression Questionnaire and Self-Esteem Measure

Depressive symptoms were measured using Zung’s (40) self-rating depression scale. This consists of 20 questions (10 positively worded and 10 negatively worded) assessing common symptoms of depression: mood disturbance (low mood, weeping), anhedonia (loss of interest or pleasure), physiological changes (trouble sleeping, constipation, weight loss), psychomotor changes (restlessness, tiredness), and anxiety (heart rate, irritability).

Participants rate each statement on a 4-point scale, indicating whether the symptom had been experienced “a little of the time”, “some of the time”, “a good part of the time”, or “most of the time” during the “past several days”. Scores range from 20 to 80, with 20–49 considered “normal range”, 50–59 “mildly depressed”, 60–69 “moderately depressed”, and 70 and above “severely depressed” (40).

We measured self-esteem using an adapted single-item self-rated question, which has been validated against Rosenberg’s (20) 10-item scale (42). Participants were asked “How would you describe your overall self-esteem?” using a sliding scale from 0 “very low” to 100 “very high”.

### Analyses

All analyses used linear regression models. In all regression analyses, we employed robust fits (to reduce the influence of outliers), and all effects were tested two-tailed. This was conducted using MATLAB [Version R2017b, linear regression model (robust fit)], which uses a bisquare weighting function. All variables were standardized where possible (except for categorical variables, e.g., gender, education level, depression group).

**Figure 2:**
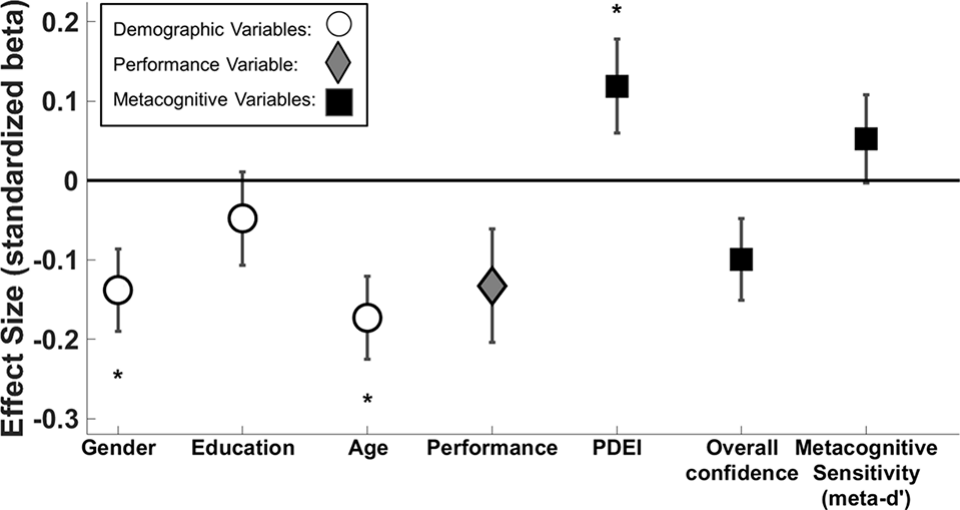
Standardized beta coefficients ( ± standard error) of predictors of depression score. White circle markers indicate demographic variables, the gray diamond indicates perceptual performance (d’ across both tasks), and black squares indicate metacognitive variables. We demonstrate significant effects of gender (β = -0.138, p = 0.008), age (β = -0.173, p = 0.001), and PDEI (β = 0.119, p = 0.045). Performance (d’ across both tasks) and overall confidence narrowly missed significance (β = -0.132, p = 0.063; β = -0.099, p = 0.056, respectively). *p < 0.05.

Regression model 1 – dependent variable: depression score; predictor variables: demographic variables (age, gender, education level), performance variables (d’, objective evidence strength, performance at higher postdecision evidence strength), and metacognitive variables (meta-d’, overall confidence, PDEI) (**Figure 2**).

Regression model 2 – dependent variable: overall confidence; predictor variables: group (depressed/nondepressed), age, gender, performance (d’), and PDEI.

Regression model 3 – dependent variable: PDEI; predictor variables: group (depressed/nondepressed), age, gender, performance (d’), and overall confidence.

Regression model 3a – dependent variable: confirmatory PDEI; predictor variables: group (depressed/nondepressed), age, gender, performance (d’), and overall confidence.

Regression model 3b – dependent variable: disconfirmatory PDEI; predictor variables: group (depressed/nondepressed), age, gender, performance (d’), and overall confidence.

Regression model 4 – dependent variable: self-esteem; predictor variables: demographic variables (age, gender, education level), performance variables (d’, objective evidence strength, performance at higher postdecision evidence) and metacognitive variables (meta-d’, overall confidence, PDEI) (**Figure 5**).

A subset of covariates from model 1 was not included in models 2–3b after determining they were not associated with depression score. For completeness, we repeated the analysis of models 2–3b with the full set of covariates to ensure that all findings remained unchanged. We checked for multicollinearity of all multiple regressions by calculating the variance inflation factor for each predictor, which was <2 for all regressions and predictors and below a standard cutoff value of 10 (43).

#### Calculation of Confidence Bias and Metacognitive Sensitivity

Confidence bias was calculated as the mean confidence rating of all trials of the confidence task and reflects an individual’s tendency to use higher or lower confidence ratings regardless of their performance.

To measure metacognitive sensitivity (the extent to which participants adjust their confidence judgments following correct or incorrect decisions) we calculated meta-d’ (44). This is based on signal detection theory and is a standard metric for assessing metacognitive sensitivity (2). The advantage of using meta-d’ is that it is not influenced by a person’s general propensity to report their confidence as higher or lower.

To estimate meta-d’ for each subject, we used a Bayesian estimation scheme (45) using the nonhierarchical version of the model.

#### Calculation of Postdecision Evidence Integration

PDEI was measured as the increase in confidence caused by postdecision (confirmatory) evidence when subjects were initially correct and the decrease in confidence caused by postdecision (disconfirmatory) evidence when subjects were initially incorrect. To this end, for each participant, we constructed a trial-by-trial linear model of data pooled across both tasks. In this model, confidence was the dependent variable, and the following predictors were entered: accuracy (correct = 1, incorrect = -1), postdecision evidence strength (confidence task = 0, low postdecision evidence = 1, high postdecision evidence = 2), and the critical accuracy × postdecision evidence strength interaction term. This interaction term quantifies the extent to which confidence increases on correct trials and decreases on error trials as postdecision evidence strength increases. This forms a summary measure of sensitivity to additional evidence (PDEI).

#### Depression Score and Self-Esteem Measure

Because of fewer participants scoring at the higher end of the depression questionnaire, we conducted a log linear transformation on depression questionnaire scores to reduce positive skew. This transformed variable was used for all subsequent analysis.

We used the arcsin transformation to reduce negative skew in our self-esteem scores, and this variable was used in all subsequent analyses.

## Results

### Overall Performance and Confidence Reports

Following the staircase procedure (in which participants’ performance was staircased to 71% accuracy), participants performed on average at 73.1% accuracy with a range of 60% to 84.9%.

Participants’ mean confidence in their decisions on the confidence task (task 1) was 75.6% (SD = 0.11) when correct and 65.8% (SD = 0.12) when incorrect. Postdecision evidence (displayed in task 2) had the expected effect on confidence ratings. For correct choices, mean confidence increased to 75. 8% (SD = 0.11) following low postdecision evidence strength and increased to 85.1% (SD = 0.11) following high postdecision evidence strength. For incorrect choices, confidence lowered to 53.4% (SD = 0.15) following low postdecision evidence strength and 38.4% (SD = 0.19) following high postdecision evidence strength. This shows that participants adjusted their confidence accordingly when shown further evidence after making their decision.

### Overall Confidence, Metacognitive Sensitivity, and Depressive Symptoms

#### Depression as a Continuous Variable

First, we constructed a multiple linear regression model (Regression model 1; **Figure 2**) to assess whether depressive symptoms were associated with lower overall confidence and better metacognitive sensitivity.

The association between overall confidence and depressive symptoms narrowly missed significance (β = -0.099, p = 0.056), and it was further weakened when controlling for self-esteem (β = 0.009, p > 0.1).

There was no association between metacognitive sensitivity (meta-d’) and depressive symptoms (β = 0.052, p > 0.1).

#### Depression as a Categorical Variable

Participants’ depression scores ranged from 20 to 77, with an average score of 37.04. Using a cutoff of 50 [recommended by Ref. (40)], 57 participants (∼14% of our sample) met criteria for at least mild depression. The mean depression score in the depressed group was 56.66 (SD = 6.21) and in the nondepressed group was 33.92 (SD = 8.07).

To investigate the difference in overall confidence between depression groups, we constructed a multiple linear regression model (Regression model 2). We found no significant association between group and overall confidence (p = 0.244) or any other variable (all p > 0.05).

### Postdecision Evidence Integration and Depressive Symptoms

#### Depression as a Continuous Variable

We used the multiple linear regression reported above (Regression model 1; **Figure 2**) to also assess the relationship between PDEI and depressive symptoms.

PDEI was significantly associated with depression score (β = 0.119, p = 0.045), meaning that participants with higher depressive symptoms were more sensitive to new information, adjusting their confidence ratings to a greater extent.

#### Depression as a Categorical Variable

**Figure 3:**
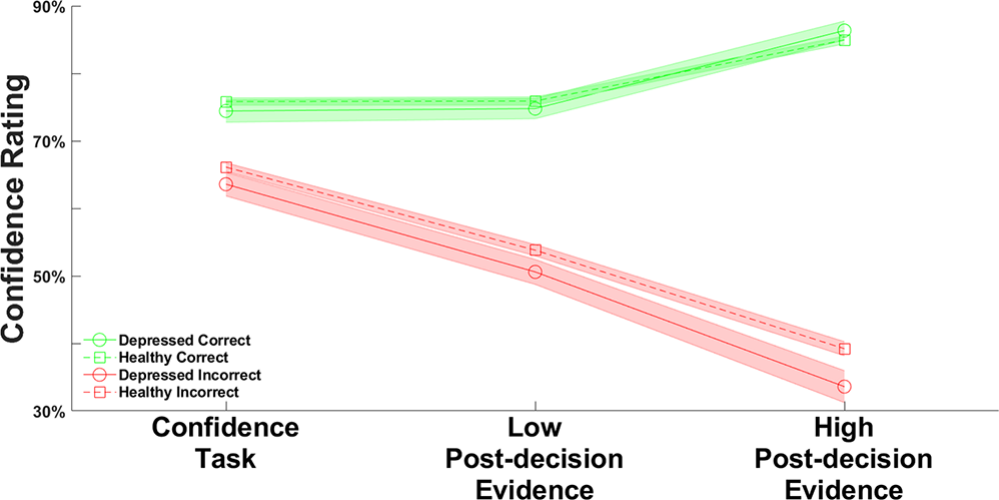
Average confidence rating across the confidence and postdecision evidence integration (PDEI) tasks, separated by group. Green lines show confidence when participants were correct; red lines show confidence when participants were incorrect. Dotted lines indicate nondepressed participants (scoring less than 50 on the depression scale); solid lines indicate depressed participants (scoring at least 50 on the depression scale). The shaded areas indicate the standard errors of the mean. In linear regression analysis, we found a significant association between group (depressed/nondepressed) and PDEI (β = 0.361, p = 0.005).

To investigate the difference in PDEI between groups, we conducted a linear regression (Regression model 3). Consistent with the analysis using depression score as a continuous variable, we found a significant positive association between PDEI and group (β = 0.124, p = 0.005). This confirms that after receiving postdecision evidence, depressed individuals adjust their confidence more than nondepressed individuals (**Figure 3**).

We then used linear regression to investigate PDEI separately for when participants received confirmatory or disconfirmatory evidence. First, we entered confirmatory evidence integration as the dependent variable (Regression model 3a). We found that group was positively associated with postdecision integration of confirmatory evidence (β = 0.094, p = 0.019), such that depressed participants exhibited a greater boost in confidence when receiving postdecision evidence after correct judgments. Second, we entered disconfirmatory evidence integration as the dependent variable (Regression model 3b). We found again that group was positively associated with postdecision integration of disconfirmatory evidence (β = 0.093, p = 0.033), such that depressed participants exhibited a greater reduction of confidence when receiving postdecision evidence after incorrect judgments. This indicates that the increased incorporation of postdecision evidence in depressed subjects is not a valence effect (e.g., depressed subjects only incorporating disconfirmatory evidence) but a general characteristic of postdecision processing.

**Figure 4:**
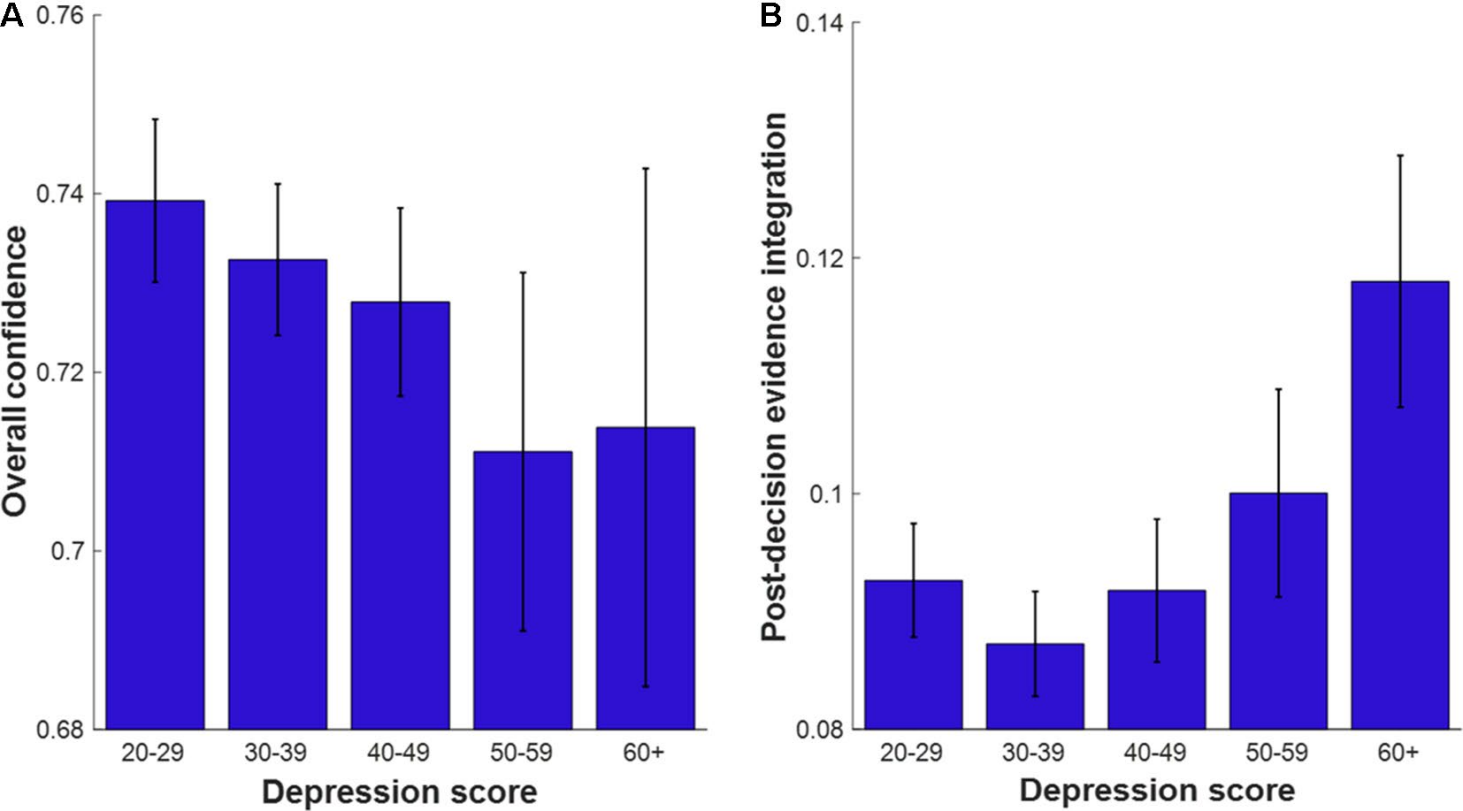
Metacognitive variables plotted as a function of depression score in five bins (50 is the recommended cutoff classifying mild depression). Error bars indicate the standard errors of the mean. **(A)** Mean Overall confidence across depression bins. **(B)** Mean Postdecision evidence integration score across depression bins. Ns per bin: depression score 20–29 (n = 120), 30–39 (n = 135), 40–49 (n = 104), 50–59 (n = 41), 60+ (n = 16).

To further visualize these associations, we binned data into five categories according to depression score (20–29, 30–39, 40–49, 50–59, and 60+). **Figure 4A, B** show the mean overall confidence and PDEI scores, respectively, across the five categories.

### Sensitivity Analysis

When performing the analyses with depression as a categorical variable (models 2–3b), we did not include education or performance at the higher staircase value as predictors because they were not associated with depression score in regression model 1. However, for consistency with all other analyses in this study, we repeated these analyses with the full set of covariates (see regression model 1). This had little influence on the results: depression group was not associated with overall confidence (β = -0.059, p = 0.235), but it was associated with PDEI (β = 0.107, p = 0.015), both for confirmatory (β = 0.090, p = 0.025) and disconfirmatory (β = 0.079, p = 0.067) evidence.

### Demographics, Performance Variables, and Depressive Symptoms

Using regression model 1, we could also investigate the relationship between depressive symptoms and demographics and performance variables. As expected, we found that age and gender were significantly associated with depressive symptoms (age β = -0.173, p = 0.001; gender β = -0.138, p = 0.008). Younger and female participants had higher depressive symptoms, consistent with a large body of prior work (46, 47).

There was a negative association between performance (d’) and depression, which narrowly missed significance (r = -0.132, p = 0.063), underscoring the importance of controlling for this variable in the analyses. However, we note that this effect is in the opposite direction to the association found between depressive symptoms and PDEI. If depression was associated with a generalized insensitivity to evidence, then we would expect that more depressed individuals would perform worse and show less PDEI. On the contrary, despite the weak association with worse performance, we find that depression is associated with greater PDEI, suggesting a specific change in metacognitive evaluation that cannot be explained by performance differences.

### Metacognitive Function and Self-Esteem

As expected, self-esteem was strongly negatively associated with depression scores (r = -0.678, p < 0.001).

**Figure 5:**
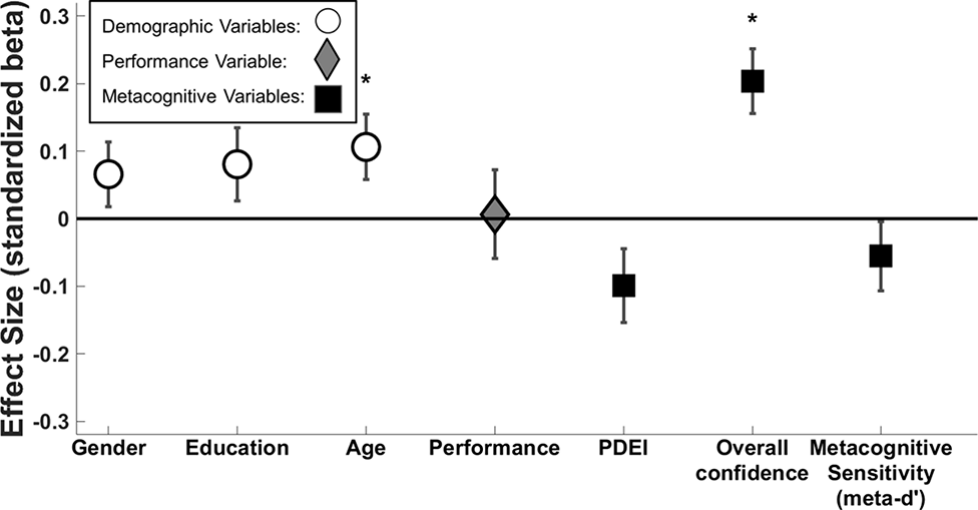
Standardized beta coefficients ( ± standard error) of predictors of self-esteem score. White circle markers indicate demographic variables, the grey diamond indicates performance (d’ across both tasks) and black squares indicate metacognitive variables. We demonstrate significant effects of age (β = .106, p = .028) and overall confidence (စβ = .203, p = < .001). *p < .05.

To investigate the relationship between self-esteem and metacognitive function, we conducted a linear regression (Regression model 4; **Figure 5**).

Overall confidence was significantly positively associated with self-esteem (β = 0.203, p < 0.001). This effect remained significant when controlling for depression score (β = 0.109, p = 0.002). There were no significant associations between self-esteem and the other measures of metacognitive function (meta-d’ p = 0.279 and PDEI p = 0.070).

We also found that age was a significant predictor of self-esteem (β = 0.106, p = 0.028), with younger participants scoring lower.

**Figure 6:**
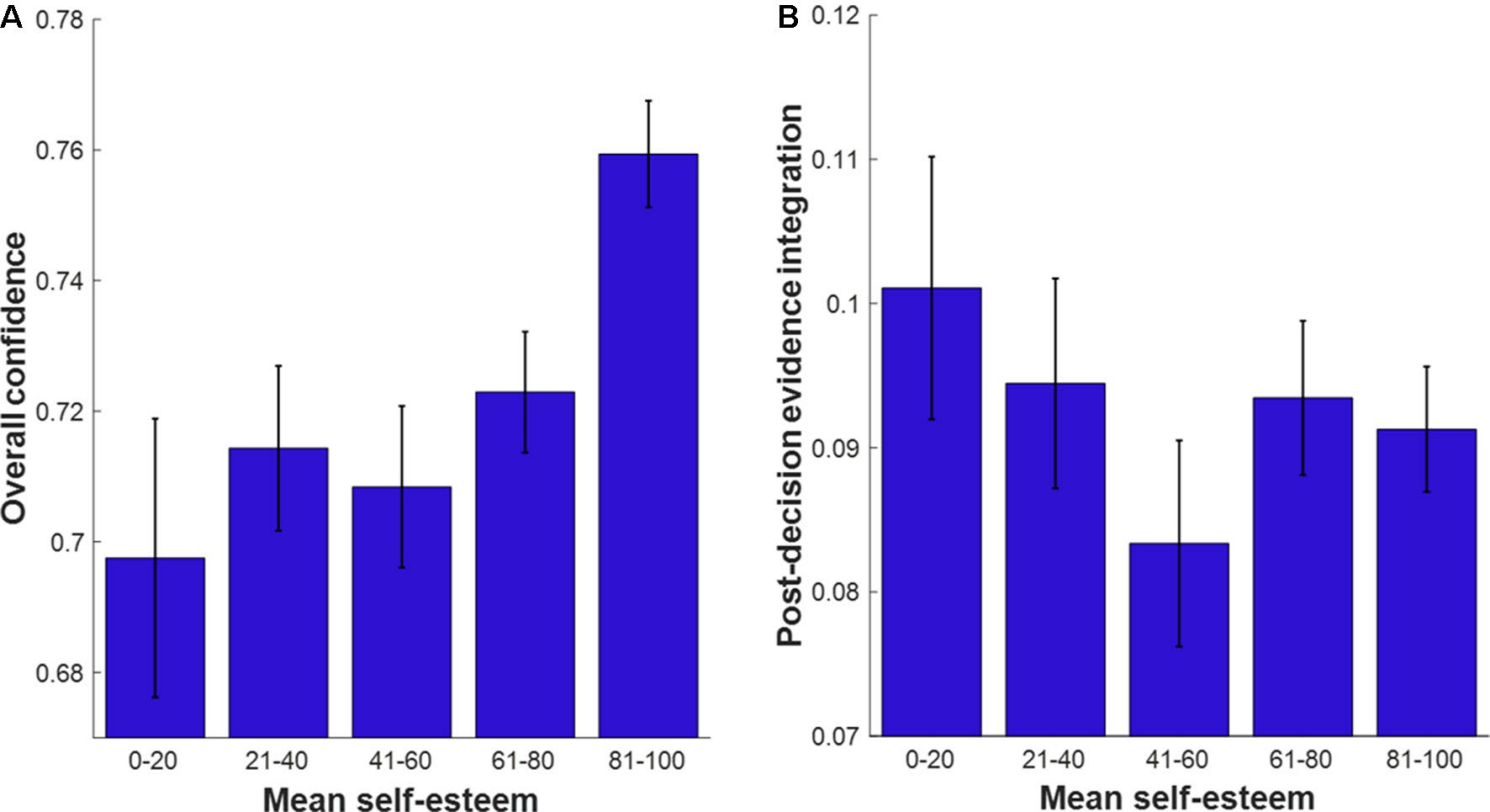
Metacognitive variables as a function of self-esteem score in five bins. Error bars indicate the standard errors of the mean. **(A)** Mean overall confidence plotted as a function of self-esteem bins. **(B)** Mean postdecision evidence integration plotted as a function of self-esteem bins. Ns per bin: Self-esteem score 0–20 (n = 44), 21–40 (n = 59), 41–60 (n = 51), 61–80 (n = 111), 81–100 (n = 151).

To further visualize these associations, we binned data into five categories according to self-esteem score (0–20, 21–40, 41–60, 61–80, 81–100). **Figure 6A, B** show mean overall confidence and PDEI score, respectively, across the five categories.

## Discussion

In a large unselected online sample, we investigated the association between metacognitive function (overall confidence, metacognitive sensitivity, and PDEI), depressive symptoms, and self-esteem.

We identified a marginal negative association between depressive symptoms and overall confidence during perceptual decision making. However, when comparing depressed and nondepressed groups, we did not observe any significant difference in overall confidence. Thus, we observed only weak evidence for our first hypothesis, derived from Rouault et al. (21), that depressive symptoms would be associated with lower metacognitive confidence.

We also did not detect any significant association between depressive symptoms and metacognitive sensitivity (meta-d’), even though in the current data set, meta-d’ and PDEI (which was associated with depressive symptoms, see below) are positively correlated (31). Thus, we did not confirm our second hypothesis that depressive symptoms would be associated with better metacognitive sensitivity.

Instead, depressive symptoms were associated with greater integration of postdecision evidence, meaning that more depressed participants adjusted their confidence more (i.e., they were more likely to change their mind) in the face of new evidence having made a decision. This pattern was also evident in a categorical analysis of depression: participants who met a threshold for at least mild depression had greater PDEI scores than participants scoring in the nondepressed range. Interestingly, the degree of PDEI was increased for both confirmatory and disconfirmatory evidence in depressed participants (albeit the association with disconfirmatory PDEI narrowly missed significance in a sensitivity analysis), indicating a generally heightened sensitivity to new evidence following a decision rather than a biased integration of negative information.

It is possible that the integration of postdecision evidence might act as a more sensitive experimental marker of self-evaluation than metacognitive sensitivity (meta-d’), which relies on endogenous fluctuations in confidence.

Our self-esteem measure was more closely related to differences in overall confidence than depressive symptoms. Overall confidence was positively associated with self-esteem and, despite the correlation between depressive symptoms and self-esteem, this association remained significant when controlling for depression score. We did not observe a significant association between self-esteem and metacognitive sensitivity or PDEI.

### Depressive Symptoms Were Associated With Greater Postdecision Evidence Integration

Depressed participants adjusted their confidence more in the face of new evidence, and this effect was exacerbated at higher evidence strengths. In our task, increased PDEI was adaptive and resulted in more accurate evaluations of participants’ decisions. However, there was no association between depressive symptoms and earnings, arguing against the notion that depressed participants were simply more motivated to win money and thus adjusted their confidence more to try to do so. Instead, this pattern is consistent with a depressive realism account (8).

Another way to interpret PDEI is in terms of changes of mind. The study of postdecisional evaluation may pave the way toward an explanation of symptoms, such as indecisiveness. Interestingly, participants in the depressed group adjusted their confidence more both when they were correct (and received confirmatory postdecision evidence) and when they were incorrect (and received disconfirmatory postdecision evidence). This symmetry argues against an influence of valence in changes of mind, for example, an oversensitivity to disconfirmatory evidence or an inability to integrate confirmatory evidence.

Importantly, by adjusting their confidence more in the face of postdecision evidence, participants in the depressed group were performing better than participants in the nondepressed group. This shows the interesting complexities in understanding the mechanisms underpinning depressive symptoms—what appears as adaptive in one task may in fact lead to maladaptive decision making in other settings. However, further evidence is needed with more direct self-report measures of indecisiveness to disentangle the contribution of metacognitive confidence to this symptom.

### Overall Confidence Was Better Explained by Self-Esteem Than Depressive Symptoms

We found a weak association between depression score and overall confidence that narrowly missed significance (p = 0.056) and was weakened when controlling for self-esteem (p > 0.1). Rouault et al. (21) found a significant association between depression score and overall confidence in experiment 1 but not in experiment 2. It is important to note, however, that the focus of the current study was on depressive symptomatology in isolation (as measured with the Zung scale), whereas in Rouault et al. (21), the strongest relationships with metacognition were observed for a factor that cross-cut elements of both anxiety and depression. Specifically, when using factor analysis to cluster symptoms independently of questionnaire of origin, Rouault et al. (21) found a strong association between their anxious–depression factor and overall confidence. This pattern of results raises the possibility that the lower confidence found in relation to greater anxious–depression factor scores in Rouault et al. (21) may be driven more by anxious than depressive symptoms.

Self-esteem showed a strong association with overall confidence in performance, as derived from the average of trial-by-trial ratings. This suggests that self-esteem may be closely related to concepts of self-efficacy (i.e., overall beliefs about self-performance). Notably, self-esteem was not related to either metacognitive sensitivity (meta-d’) or PDEI, suggesting that it tracks overall beliefs about the probability of success, rather than affecting postdecisional monitoring. Future research should address whether this relationship occurs across multiple task paradigms, exploring different dimensions of self-esteem, for example, self-efficacy versus self-liking (48).

### Limitations and Future Directions

Several limitations to this study merit comment. First, we recruited and tested participants from an unselected online sample. This meant that we recruited relatively few participants who scored over the cutoff for mild depression (cutoff score 50, ∼14% of our sample), and the mean depression score of this group was quite mild (M = 56.66). Second, we were only able to obtain a single questionnaire measure of depressive symptoms (40) and a single-item measure of self-esteem (42) because of time constraints and concerns about sustained attention in online experiments (49). Finally, we were unable to identify participants who met clinical criteria for a major depressive episode using this scale (40). We also did not collect any details on previous episodes or comorbidities, life events leading up to the time of the experiment or current psychiatric treatment. It is possible, for instance, that people who have suffered negative shocks to self-efficacy (such as social or professional rejections) may have both higher depression scores and lower global metacognitive evaluations (21).

Therefore, future research should confirm these findings by comparing well-characterized clinically depressed patient populations with matched healthy controls. Collecting detailed psychiatric histories and measures of life events would allow the assessment of relationships between these variables and metacognitive measures. It would also be worthwhile to investigate symptom profiles in more detail to examine how specific symptoms (e.g., indecisiveness) relate to PDEI.

Participants in our study were given a monetary incentive to rate their confidence as accurately as possible. The monetary reward was calculated using a quadratic scoring rule that means that participants earn the most points when maximally confident and correct or minimally confident and incorrect. When comparing depressed and nondepressed groups, one possible concern is that this incentive might not have had the same value across the participants; for example, depressed participants might be less motivated to win money because of disrupted motivation. However, two factors lessen this concern: 1) depressed participants did not earn significantly less money than nondepressed participants; 2) our main finding that depressed participants adjusted their confidence more following further evidence (PDEI) would indicate that, if anything, depressed participants considered the accuracy of their confidence ratings more carefully than nondepressed participants.

Another potential concern is that this study was carried out as part of a replication experiment within a previous study on political attitudes (31), which could raise the Type I error rate because we did not correct for multiple comparisons in relation to the other questionnaires in the study (although here our focus was on associations with depressive symptoms, not political attitudes). Therefore, these results should be treated with caution until the study is independently replicated.

## Conclusion

We have identified small but significant shifts in metacognitive function associated with higher depressive symptoms and lower self-esteem. We found some evidence that depressive symptoms were associated with lower overall confidence [as shown in Ref. (21)], but we were not able to replicate the finding that depressive symptoms were associated with greater metacognitive sensitivity (meta-d’) (21). We found that depressive symptoms were associated with greater PDEI, and that a self-esteem measure was better able to account for differences in overall confidence than depression score. We were able to demonstrate this in a large sample of unselected participants recruited online, highlighting the potential of this method of recruitment in psychological experiments (50). However, this method inevitably has some limitations, particularly in relation to the characterization of symptoms. Future studies should examine PDEI in well-characterized patient populations with more comprehensive measures of symptoms.

## Data Availability

The datasets generated for this study are available on request to the corresponding author.

## Ethics Statement

Participants gave informed consent and the study was approved by the Research Ethics Committee of University College London (study number 1260-003).

## Author Contributions

All four authors designed the experiment and contributed to the writing of the manuscript. MR collected the data. MM-P analyzed the data and wrote the first draft of the manuscript.

## Funding

The Wellcome Centre for Human Neuroimaging is supported by core funding from the Wellcome Trust (203147/Z/16/Z). S.M.F. is supported by a Sir Henry Dale Fellowship jointly funded by the Wellcome Trust and the Royal Society (206648/Z/17/Z)

## Conflict of Interest Statement

The authors declare that the research was conducted in the absence of any commercial or financial relationships that could be construed as a potential conflict of interest.
